# Development and validation of a self-administered questionnaire to estimate the distance and mode of children’s travel to school in urban India

**DOI:** 10.1186/s12874-015-0086-y

**Published:** 2015-10-28

**Authors:** Shailaja Tetali, Phil Edwards, G. V. S. Murthy, I. Roberts

**Affiliations:** Indian Institute of Public Health, Plot No # 1, ANV Arcade, Amar Co-op Society, Madhapur, Kavuri Hills, Hyderabad, 500033 India; London School of Hygiene and Tropical Medicine, Keppel Street, London, WC1E 7HT UK

**Keywords:** Active transport, Questionnaire development, Validity, Distance, Mode, Hyderabad, India

## Abstract

**Background:**

Although some 300 million Indian children travel to school every day, little is known about how they get there. This information is important for transport planners and public health authorities. This paper presents the development of a self-administered questionnaire and examines its reliability and validity in estimating distance and mode of travel to school in a low resource urban setting.

**Methods:**

We developed a questionnaire on children’s travel to school. We assessed test re-test reliability by repeating the questionnaire one week later (*n* = 61). The questionnaire was improved and re-tested (*n* = 68). We examined the convergent validity of distance estimates by comparing estimates based on the nearest landmark to children’s homes with a ‘gold standard’ based on one-to-one interviews with children using detailed maps (*n* = 50).

**Results:**

Most questions showed fair to almost perfect agreement. Questions on usual mode of travel (κ 0.73- 0.84) and road injury (κ 0.61- 0.72) were found to be more reliable than those on parental permissions (κ 0.18- 0.30), perception of safety (κ 0.00- 0.54), and physical activity (κ -0.01- 0.07). The distance estimated by the nearest landmark method was not significantly different than the in-depth method for walking , 52 m [95 % CI -32 m to 135 m], 10 % of the mean difference, and for walking and cycling combined, 65 m [95 % CI -30 m to 159 m], 11 % of the mean difference. For children who used motorized transport (excluding private school bus), the nearest landmark method under-estimated distance by an average of 325 metres [95 % CI −664 m to 1314 m], 15 % of the mean difference.

**Conclusions:**

A self-administered questionnaire was found to provide reliable information on the usual mode of travel to school, and road injury, in a small sample of children in Hyderabad, India. The ‘nearest landmark’ method can be applied in similar low-resource settings, for a reasonably accurate estimate of the distance from a child’s home to school.

**Electronic supplementary material:**

The online version of this article (doi:10.1186/s12874-015-0086-y) contains supplementary material, which is available to authorized users.

## Background

About 300 million children travel to school every day in India [1]. However, little is known about how they get there. Research from high-income countries shows that children are more likely to use motorised transport if the distance to school is greater [2, 3]. Other factors associated with motor vehicle use are age [4–6], gender [2, 7], parental concerns about safety [8, 9], physical infrastructure, and weather conditions [10]. We do not have similar information in India that would help us better understand children’s school travel. There is evidence to suggest that everyday travel by walking and cycling is associated with positive health benefits for children [11, 12]. We need information on children’s travel to school in India to understand the public health impacts of these journeys. Developing methods to measure children’s travel to school for use in low resource settings is therefore important.

A range of methods have been used in high-income countries to measure distance from home to school: Geographical Information Systems (GIS) [10, 13]; Geographical Positioning Systems (GPS) [14]; travel time [15]; or the ‘straight-line’ between school and home [4, 16]. Distances have been calculated using the shortest route possible along the road network [17] or by asking children to draw their routes to school on image maps which were then digitalized and measured, using GIS [18]. In many low resource settings in India, postcodes and addresses often do not identify dwellings and cannot be used to reliably estimate distance to school.

This paper presents the development and testing of a self-administered questionnaire on children’s travel to school. This is part of a larger study that aims to estimate the distribution of children’s mode of travel to school in Hyderabad (Telangana, India), a city with a population of almost 8 million [19]. A cross-sectional survey is planned to collect data from about 6,000 school children aged 11–14 years, which will be incorporated into a spreadsheet model of the public health impacts of school travel. Accurate estimates of distances and modes of travel by children in Hyderabad is an essential component of the study. The objective of this study was to develop a self-administered questionnaire and examine its reliability and validity in estimating distance and mode of travel to school.

## Methods

We developed a questionnaire for use in children aged 11–14 years, as this is typically an age when children may be expected to travel independently [20]. In school terminology, it refers to children in grades 6–9.

### Questionnaire development

We searched the literature to identify questions that could be applied in the context of a low resource setting like India (see Additional file 1) [8, 21]. We originally identified about 25 items from previously published work on children’s independent travel and adapted them for the Indian context [20]. We conducted a focus group with four public health experts to discuss the appropriateness of the questions. We included a question that asked children about the nearest landmark to their home and used this to estimate the distance from home to school. The final questionnaire (Additional file 3) had 21 multiple choice items: four on demographics, nine on mode of travel and travel during dry or wet weather, two items on parental permissions for independent travel, three on children’s perceptions of safety, including road traffic injuries, and three items on physical activity after school. These questions were included because of our interest in children’s commuting to school in Hyderabad, and its impacts on health.

### Reliability studies

We assessed the comprehension of the questionnaire by focus group discussions among children aged 12–15 years, to assess the suitability of questions for the target age. We piloted the questionnaire in a private school (run by a Society/Trust, without government aid) [22] with 12 children of grade nine, noting all requests for clarifications. For assessing the reliability of the questionnaire, we distributed Telugu translated questionnaires to children in grade eight of a government school (*n* = 61) and conducted a re-test one week later. Telugu is the first language spoken by about 80 million people in India and is the local language in Hyderabad, where this study was conducted. We back-translated the questionnaire, to ensure the correct interpretation of the questions. We conducted a second reliability study in another government school (*n* = 68). We administered questionnaires using pencil-and-paper methods and read out each question, allowing plenty of time for marking the responses.

### Validation of estimated distance

We assessed the validity of the distance estimates based on the ‘nearest landmark to home’ method, by comparing with a ‘gold standard’ measure, based on in-depth one-to-one interviews with 50 school children in grades 7, 8 and 9, using detailed maps of their neighbourhood and routes to school. The class teacher randomly selected children using each mode of transport. Fifty children, with 56 % (*n* = 28) females participated in the ‘in- depth interview’ method. The distribution of school-type was government (30 %, *n* = 15); semi-private (26 %, *n* = 13) and private (44 %, *n* = 22).

#### Gold standard in-depth interview method

*Google Earth* [23] was installed on a laptop computer, with a ‘place mark’ on the map corresponding to the school. We visited one school of each type (i.e. Government, semi-private and private). After a brief orientation, each child traced the route from his/her home to school, using a finger. Each route was recorded in *Google Earth*. We used the *‘Play tour’* viewing mode for children to see and confirm their routes to school, as well as the distance travelled.

#### Nearest landmark method

Using *Google maps*, [24] the ‘nearest landmark’ information of each of the 50 children was entered in the ‘*from*’ box and the school address in the ‘*to*’ box. The ‘*give directions*’ button gave a suggested route and corresponding distance. [Example screenshots of both methods are shown in the Additional file 2].

### Statistical analysis

STATA 12 (Stata Corp, College Station, Texas) was used for statistical analysis. For the reliability studies, agreement was assessed for each question using the kappa statistic. Standard categories were used for interpreting agreement (i.e. κ >0.81 ‘almost perfect’ agreement; κ 0.61- 0.80 ‘substantial’ agreement; κ 0.41- 0.60 ‘moderate’ agreement; κ 0.21- 0.40 ‘fair’ agreement; κ 0.01 - 0.20 ‘slight’ agreement; κ 0.00 ‘less than chance’ agreement) [25]. The difference between the distances estimated by the two methods was plotted against the average of the two distances using a Tukey/Bland Altman plot [26]. Limits of agreement were calculated as the mean difference ±1.96 × SD, within which 95 % of the observed differences would be expected to lie. A paired sample *t*-test was used to assess whether the bias (mean difference) was statistically different from zero, where statistical significance was at the 5 % level.

Prior permissions were obtained from the Hyderabad District Education Office. The participating school principals gave verbal consent on behalf of the children, and parents/guardians were informed of the study. Ethics committee approved consent being taken only from the school principal. Ethical approvals were secured from the London School of Hygiene and Tropical Medicine, London, UK, and the Indian Institute of Public Health, Hyderabad, India.

## Results

### Questionnaire development

The pilot confirmed that the questionnaire could be completed in 15–20 minutes. After the first reliability study, definitions were added for *exercise, main roads*, and *feeling safe*.

### Reliability studies

Table 1 shows the results of the reliability studies. There were 61 children in the first reliability study and 68 children in the second. Fifteen children absent during the re-tests were removed from analysis. There was perfect agreement for age, sex and name. Almost all children (67 out of 68) wrote the same landmark in the test and re-test. The first reliability study showed ‘substantial’ or ‘moderate’ agreement in 69 % (11/16) questions; ‘fair’ agreement in 6 % (1/16) questions and ‘slight’ agreement in 25 % (4/16) questions. The second reliability study showed ‘almost perfect’ agreement in 11 % (2/17) questions, ‘substantial or moderate’ agreement in 41 % (7/17) questions, and ‘fair’ agreement in 23 % (4/17) questions. Questions on usual mode of travel to school showed ‘substantial’ to ‘almost perfect’ agreement. The question on road injury showed ‘substantial’ agreement in both the reliability studies. Questions on parental permissions for independent travel, perceptions of safety, and physical activity after school were shown to be less reliable.

**Table 1:** Results of reliability studies

Questionnaire item	Questionnaire version 1 kappa	Questionnaire version 2 kappa
How did you travel to school today?	0.67	0.79
With whom did you come to school today?	0.53	0.31
How do you travel to school during a usual week?	0.73	0.75
How will you go from school to home today?	0.75	0.66
With whom will you go from school to home today?	0.58	0.58
How do you travel home during a usual week?	0.76	0.84
How would you like or wish to travel to and from school?	0.48	0.44
How do you travel to school during the rains?	0.56	0.64
How do you travel to school during hot weather?	0.66	0.88
Are you allowed by your parents to cross main roads alone?	0.18	0.24
Are you allowed by your parents to cycle on main roads alone?	0.30	0.20
How safe do you feel when you travel to and from school?	0.02	0.00
What are you worried about, during your journey to school?	0.54	0.31
During the past week, after school, on how many days did you exercise?	0.07	0.01
^a^During the past week, after school, how many hours did you exercise?	n/a	0.01
During the past week, how many Physical Training (PT) periods did you attend?	0.07	−0.01
During the past 12 months, were you injured in a road accident?	0.61	0.72
^a^Mention the nearest landmark to your home	n/a	n/a

^a^Question included only in the revised version

### Validation of estimated distance

Table 2 shows the average difference between the two methods of measurement for different modes of travel. It shows that no mean differences were statistically significant. Only one child reported coming by ‘van’ (private transport paid by parents) and was combined with ‘school bus’ (also private) for analysis. The ‘nearest landmark’ estimates were not significantly different from the ’in-depth interview’ estimates. The distance estimated by the nearest landmark method was not significantly different than the in-depth method for walking , 52 m [95 % CI -32 m to 135 m], 10 % of mean difference, and for walking and cycling combined, 65 m [95 % CI -30 m to 159 m], 11 % of mean difference. For children who travelled by school bus/van, the ‘nearest landmark’ method under-estimated the distance by approximately 2.4 km (37 % of the mean difference). For children who travelled by motorized transport excluding the school bus, the ’nearest landmark‘ method under-estimated distance by an average 325 metres [95 % CI −664 m to 1314 m], 15 % of the mean difference.

**Table 2:** Mean difference between methods by mode

Mode of travel^a^	n	Mean distance m (In-depth)	Mean difference m (In-depth - landmark)	95 % CI	Difference as % of mean distance	*P* value
Walk	20	525	−52	(−135, 32)	−9.9	0.27
Walking or cycling	23	602	−65	(−159, 30)	−10.8	0.10
Auto rickshaw	5	2309	−391	(−918, 137)	−16.9	0.10
Motorbike	8	2403	91	(−190, 371)	3.8	0.53
Car	3	5356	523	(−1464, 2510)	9.8	0.37
RTC bus (Public)	7	3640	69	(−263, 402)	1.9	0.62
School bus/ Van	4	6436	2386	(−847, 5619)	37.1	0.10
Motorized travel (excluding school bus/van)	23	2202	325	(−664, 1314)	14.8	0.17

^a^Other response categories like train were not marked by any child in this study

**Fig. 1:**
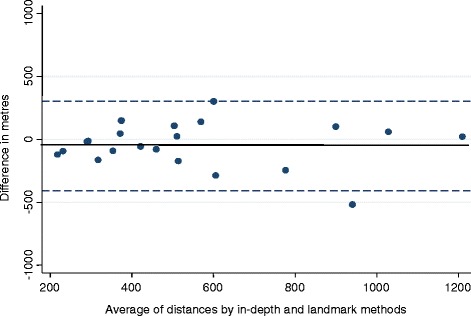
Differences between ‘in-depth interview’ and ‘nearest landmark’ methods (walking). Limits of Agreement = Mean difference +/− 1.96SD = −407 m to 304 m. Mean difference = − 52 m

Figure 1 shows the mean difference plot for walking. The dotted lines show the limits of agreement, and the solid line shows the bias (−52 m).

**Fig. 2:**
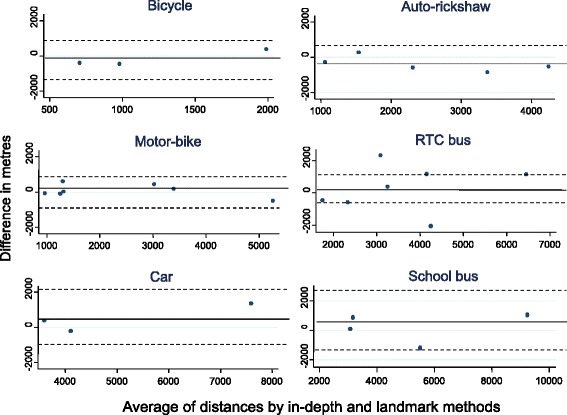
Differences between ‘in-depth interview’ and ‘nearest landmark’ methods (different modes)

Figure 2 shows the mean difference plots for different modes. The dotted lines show the limits of agreement.

## Discussion

### Principal findings

The questionnaire on children’s travel to school showed that the questions on usual mode of travel, and road injury were reliable. Distance to school measured by asking for the nearest landmark to a child’s home was found to be a valid measure of distance when compared to a method based on in-depth interviews with children. This was true for different modes of travel to school in Hyderabad, but to a lesser extent with the school bus.

### Strengths and weaknesses

Questionnaires were administered one week apart and some children’s motivation and interest may have differed between occasions, altering the quality of their responses. There was a difference in the number of children who took the test and re-test, but it is not expected that the exclusion of the absentees would influence the results. Compared to those present, absentees had similar age (12.9 vs 13.1 years, *p* = 0.09), and sex (44 % vs 47 % boys, *p* = 0.55), and prevalence of walking (74 % vs 69 %, *p* = 0.99).

Due to limited resources, we could not use objective measures of distance such as GPS. Children’s home address was not included because many urban areas in India including several localities in Hyderabad are growing rapidly. As a result, they do not have uniformly structured or geocoded searchable addresses on the web [27]. In the absence of searchable addresses, our questionnaire provides a cost-effective alternative. Reliability was assessed using written survey forms instead of ‘hand-raising’ protocols used in other studies [28].

*Google Earth* is increasingly being used in Public Health [29, 30]. We used *Google Earth* and *Google Maps* as they are freely available and easy to use, and due to a lack of access to other GIS tools. It is suggested that *Google Earth* images should be checked for accuracy [31] because they may not reflect recent changes in landscape like new urban development and recent disasters [32]. The distance from home to nearest landmark was not accounted for in this analysis, and could therefore slightly alter the distance estimated.

### Strengths and weaknesses in relation to other studies

The ‘in-depth’ method of recording children’s journeys enabled good quality data to be collected, which was the strength of this study. Other studies have relied on parent’s reports [18, 33] but we did not involve parents because of concerns about high levels of illiteracy among low-income parents in India. The kappa score for the question on “mode of travel to school today” was lower than that obtained by another study that also used pen and paper (i.e. 0.79 vs 0.98) [25]. This was perhaps because it administered the questionnaire on the same day rather than one week apart. The difference in kappa in our survey could also be due to the difference in the travel behaviour on the day of the survey.

Questions on the usual mode of travel and road injury were found to be more reliable than those on parental permissions, perception of safety, and physical activity, and this must be considered before using the questionnaire. The question on physical activity adapted from the WHO Global School Health Survey [34] was found to be especially challenging and many children asked for clarification. No evidence of bias was found in the distance estimate when walking and cycling were combined. The nearest landmark distance was slightly greater for walking, and when walking and cycling were combined, and for auto-rickshaw. Children probably take short-cut routes which *Google* may not consider. This was not the case with the school bus, which undertakes long winding routes to collect children from their homes, and does not reflect the distance from home to school that would be travelled using other modes. For all types of motorized travel, the ‘nearest landmark’ distance was shorter than the ’in-depth interview‘ distance, with the exception of auto rickshaw, perhaps due to its ability to take short-cut routes, possibly leading to traffic violations [35].

### Meaning of the study and future research

This study developed a questionnaire on mode of travel to school and a method to estimate the distance that children travel to school in Hyderabad, India. It may be used to determine whether these are journeys that could be made by walking or cycling. In the absence of searchable databases to pinpoint the home location, we used *Google Earth* and *Google Maps* to estimate distance. When we compared the ‘nearest landmark’ versus ‘in-depth’ distance, they differed by 10 % for walking and cycling. We consider this margin of error to be within acceptable limits of accuracy. For other modes like the school bus, the mean difference is higher, but this is because the school bus does not use a direct route. Future studies can therefore use the nearest landmark method to estimate the true distance that a child would walk or cycle to school. It confirms that the nearest landmark method is feasible, in the absence of GPS equipment and software, especially in low resource urban settings.

This method should be tested in rural areas, which have a different pattern of land-use. Further development of this approach, for example using factor analysis to refine the items, may also improve the questionnaire.

## Conclusions

A self-administered questionnaire was found to provide reliable information on the usual mode of travel to school, and road injury, in a small sample of children in Hyderabad, India. The ‘nearest landmark’ method can be applied in similar low-resource settings, for a reasonably accurate estimate of the distance from a child’s home to school.

## Additional files

Additional file 1:
**Appendix Screen shots of two methods of estimating distance to school.** (PDF 658 kb) Additional file 2:
**Appendix Search methods.** (DOCX 16 kb) Additional file 3:
**English version of questionnaire.** (PDF 468 kb)

## Abbreviations

GIS: Geographical Information Systems
GPS: Geographical Positioning Systems
WHO: World Health Organisation
