# Supplementary material for: Development and validation of a self-administered questionnaire to estimate the distance and mode of children’s travel to school in urban India
Source: BMC Med Res Methodol. 2015 Oct 28;15:92. doi: 10.1186/s12874-015-0086-y (PMC4625478; doi:10.1186/s12874-015-0086-y)

Appendix Screen shots of two methods of estimating distance to school

'In-depth interview' method for route to school by car (estimated distance 8.2km)

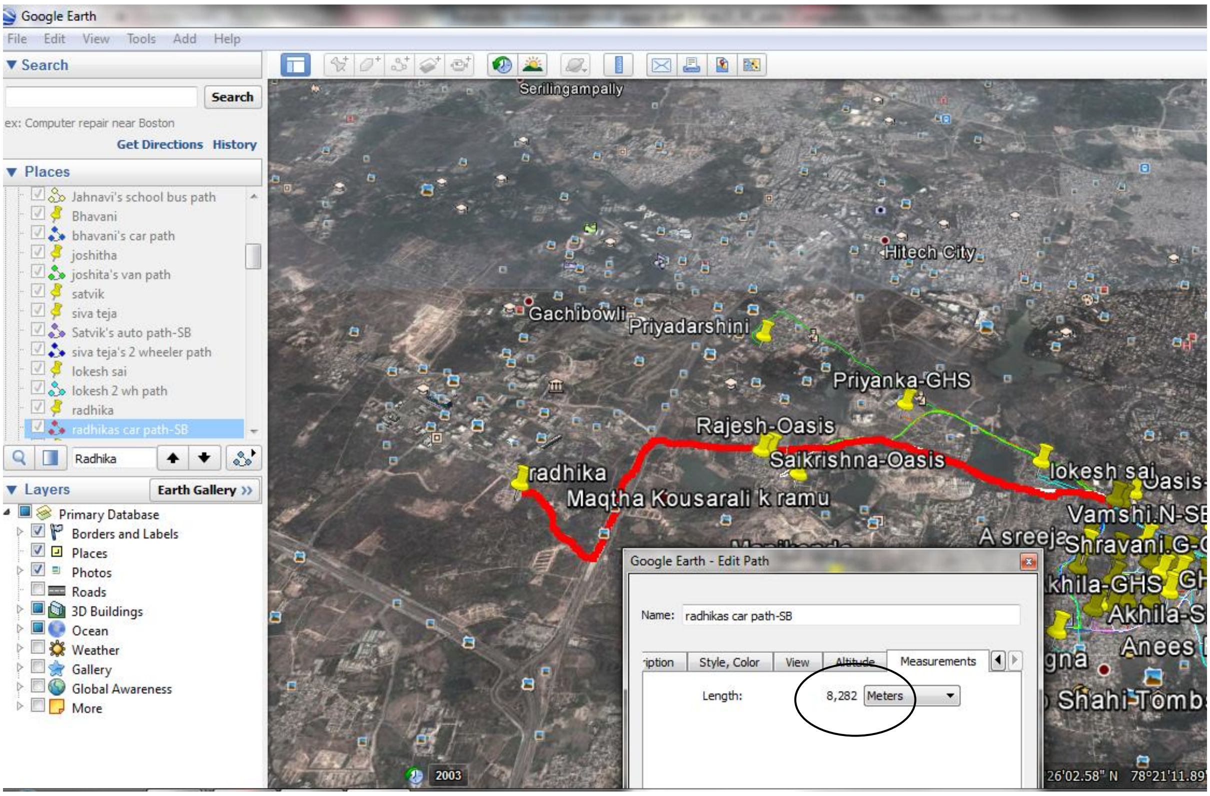

'Nearest landmark to home' method for same route to school by car (estimated distance 6.9km)

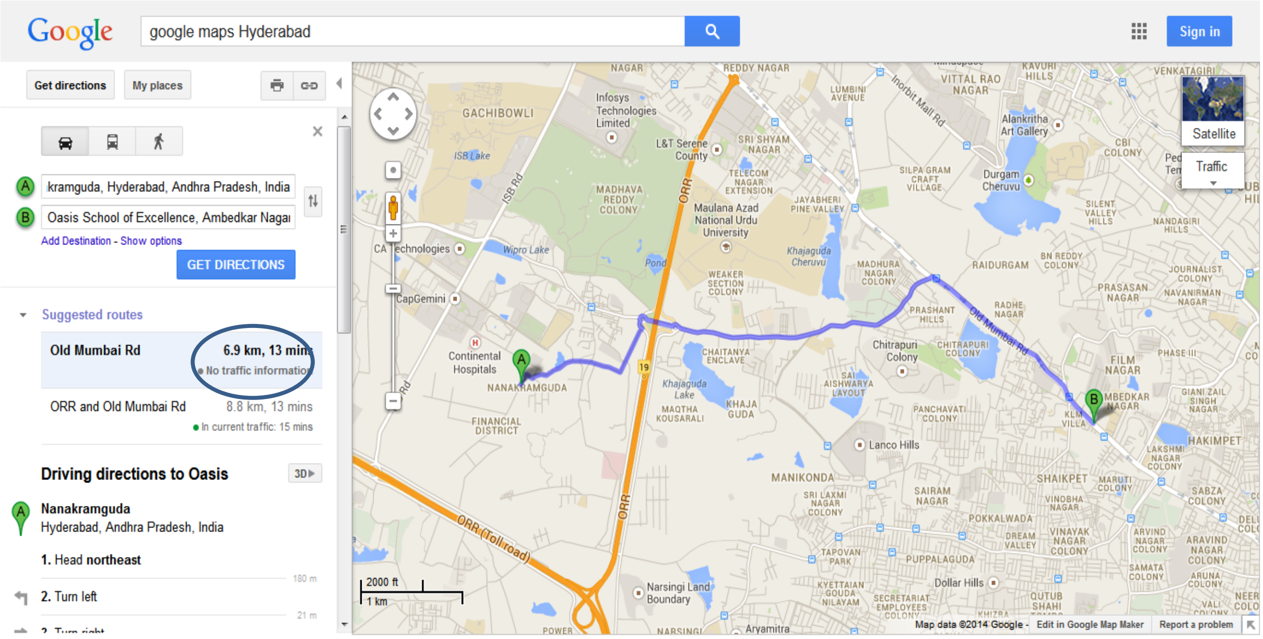

Supplement: Additional file 1: — Appendix Screen shots of two methods of estimating distance to school. (PDF 658 kb) [file 12874_2015_86_MOESM1_ESM.pdf]
