# Supplementary material for: Development and validation of a self-administered questionnaire to estimate the distance and mode of children’s travel to school in urban India
Source: BMC Med Res Methodol. 2015 Oct 28;15:92. doi: 10.1186/s12874-015-0086-y (PMC4625478; doi:10.1186/s12874-015-0086-y)
Supplement: Additional file 2: — Appendix Search methods. (DOCX 16 kb) [file 12874_2015_86_MOESM2_ESM.docx]

**Additional file 2**

**Box Search methods**

Databases searched: *Ovid, PubMed, Web of Science, Zetoc, TRID*

(1990- 2013)

Search strategy:

1. (child* OR adolesc* OR youth OR young people OR student* OR pupil* OR teenage* OR young person OR boys OR girls OR pediatri*)

2. (walk* OR active OR bicyc* OR bik* OR rid* OR cycl* OR travel* OR mode OR trip OR transport* OR commut* OR journey* OR car OR bus OR train OR auto OR rickshaw OR motorcycl* OR two wheel* OR independen*)

3. (distance OR length AND (“school”) AND (develop* countries OR rich OR high AND middle AND low income countries OR nations)

4. (question* OR tool)
