# Supplementary material for: Development and validation of a self-administered questionnaire to estimate the distance and mode of children’s travel to school in urban India
Source: BMC Med Res Methodol. 2015 Oct 28;15:92. doi: 10.1186/s12874-015-0086-y (PMC4625478; doi:10.1186/s12874-015-0086-y)
Supplement: Additional file 3: — English version of questionnaire. (PDF 468 kb) [file 12874_2015_86_MOESM3_ESM.pdf]

Survey on the Public Health Impacts of Children's Travel to School

Please answer the following questions as best as you can- there are no right or wrong answers. The answers you give will be kept private. Thank you for your help.

1.Name: ____________________

2. Age: ________ years

3. Gender: 1.Boy ☐ 2. Girl ☐

4. Home address & landmark: ____________________

Travel to School

5. How did you travel to school today?

No.	Mode of travel	From	To	Time taken (minutes)
1	Walk			
2	Cycle			
3	School bus			
4	Car			
5	2 wheeler			
6	RTC bus			
7	Auto-rickshaw			
8	Cycle-rickshaw			
9	Train			
10	Other			

6. With whom did you come to school today?

1. Parent ☐ 2. Grand-parent ☐ 3. Other children ☐ 4. Other adult ☐ 5. Alone ☐

7. How do you travel to school during a usual week?

1. Walk ☐ 2. Cycle ☐ 3. School bus ☐ 4. Car ☐ 5. 2 wheeler ☐
6. RTC bus ☐ 7. Auto ☐ 8. Rickshaw ☐ 9. Train ☐ 10. Other ☐

Travel to Home

8. How will you go from school to home today?

1. Walk ☐ 2. Cycle ☐ 3. School bus ☐ 4. Car ☐ 5. 2 wheeler ☐
6. RTC bus ☐ 7. Auto ☐ 8. Rickshaw ☐ 9. Train ☐ 10. Other ☐

9. With whom will you go from school to home today?

1. Parent ☐ 2. Grand-parent ☐ 3. Other children ☐ 4. Other adult ☐ 5. Alone ☐

10. How do you travel home during a usual week?

1. Walk ☐ 2. Cycle ☐ 3. School bus ☐ 4. Car ☐ 5. 2 wheeler ☐
6. RTC bus ☐ 7. Auto ☐ 8. Rickshaw ☐ 9. Train ☐ 10. Other ☐

11. How would you LIKE to or WISH to travel to and from school?

1. Walk ☐ 2. Cycle ☐ 3. School bus ☐ 4. Car ☐ 5. 2 wheeler ☐
6. RTC bus ☐ 7. Auto ☐ 8. Rickshaw ☐ 9. Train ☐ 10. Other ☐

12. How do you travel to school during the RAINS?

1. Walk ☐ 2. Cycle ☐ 3. School bus ☐ 4. Car ☐ 5. 2 wheeler ☐
6. RTC bus ☐ 7. Auto ☐ 8. Rickshaw ☐ 9. Train ☐ 10. Other ☐

13. How do you travel to school during HOT WEATHER?

1. Walk ☐ 2. Cycle ☐ 3. School bus ☐ 4. Car ☐ 5. 2 wheeler ☐

6. RTC bus ☐ 7. Auto ☐ 8. Rickshaw ☐ 9. Train ☐ 10. Other ☐

14. Are you allowed by your parents to cross main roads alone? (Main roads are important, busy roads with lots of traffic)

1. Always ☐ 2.Sometimes ☐ 3. Rarely ☐ 4. Never ☐

15. Are you allowed by your parents to cycle on main roads alone?

1. Always ☐ 2. Sometimes ☐ 3. Rarely ☐ 4. Never ☐ 5. I don't know cycling ☐

16. How safe do you feel when you travel to and from school? (Safe means not worried, not feeling uneasy about anything in particular)

1. Very safe ☐ 2. Fairly safe ☐ 3. Not very safe ☐ 4. Not at all safe ☐

17. What are you worried about, during your journey to school?

1. Traffic ☐ 2. Strangers ☐ 3. Being late ☐

4. Getting lost ☐ 5. Being teased ☐ 6. Nothing ☐

18. During the past week, after school, on how many days did you exercise?

(Example: running, fast walking, playing games, cycling, dancing, sports).

Do not include your PT or games period.

None ☐ 1 day ☐ 2 days ☐ 3 days ☐ 4 days ☐

5 days ☐ 6 days ☐ 7 days ☐

19. During the past week, after school, how many hours did you exercise?

Do not include your PT or games period.

None ☐ half an hour a week ☐ 1 hour a week ☐

2- 3 hours a week ☐ 4-6 hours a week ☐ 7 hours a week ☐

20. During the past week, how many PT or games periods did you attend?

None ☐ 1 period ☐ 2 periods ☐ 3 periods ☐ 4 periods ☐

5 periods ☐ 6 periods ☐ 7 periods ☐

21. During the past 12 months, were you injured in a road accident? *(An injury is when it makes you miss at least one full day of usual activities **OR** requires treatment by a doctor or nurse).*

1. Yes ☐ 2. No ☐
